# The cornea–trigeminal axis: potassium channels as master regulators of ocular surface homeostasis and sensory transduction

**DOI:** 10.3389/fcell.2026.1857722

**Published:** 2026-08-24

**Authors:** Jiali Zhang, Xinyi Chen, Jing Ji, Pengfan Chen, Jiaxin Yao, Longqian Liu, Li Tang, Xiangyi Wen

**Affiliations:** 1 Department of Ophthalmology, Department of Optometry and Visual Science, Laboratory of Optometry and Vision Sciences, West China Hospital, Sichuan University, Chengdu, Sichuan, China; 2 West China School of Medicine, Sichuan University, Chengdu, Sichuan, China; 3 Department of Ophthalmology, West China Hospital, Sichuan University, Chengdu, Sichuan, China

**Keywords:** cornea, dry eye disease, neuropathic pain, potassium channels, sensory transduction, trigeminal ganglion

## Abstract

**Introduction:**

The cornea and trigeminal ganglion (TG) form an integrated sensory network that monitors ocular surface integrity and mediates protective responses to environmental stimuli. Potassium (K^+^) channels are central regulators along this cornea–TG axis, controlling membrane excitability, sensory thresholds, epithelial repair, stromal remodeling, and inflammatory signaling.

**Methods:**

This review synthesizes current knowledge of K^+^ channel distribution, function, and dysfunction within the cornea–TG axis, based on findings from animal, ex vivo, and human tissue models.

**Results:**

In corneal epithelial and stromal cells, channels such as Kir4.1 and K_Ca_3.1 modulate membrane potential and intracellular Ca^2+^ dynamics, coordinating wound healing and fibrotic responses. In sensory neurons, K_v_, K_2P_, and K_Ca_ channels cooperate with TRP channels to integrate mechanical, thermal, and chemical inputs, setting firing thresholds and shaping nociceptive signaling. Dysregulation of these channels contributes to dry eye disease, corneal neuropathic pain, and impaired epithelial repair, highlighting their mechanistic and translational significance.

**Discussion:**

Despite advances in animal and *ex vivo* models, species-specific variability, limited human tissue validation, and a lack of cell-type resolution constrain current understanding. Future studies leveraging physiologically relevant models, selective pharmacological tools, and longitudinal analyses are critical to define dynamic K^+^ channel regulation and to guide the development of novel therapeutic strategies for ocular surface disease.

## 1 Background

**FIGURE 1:**
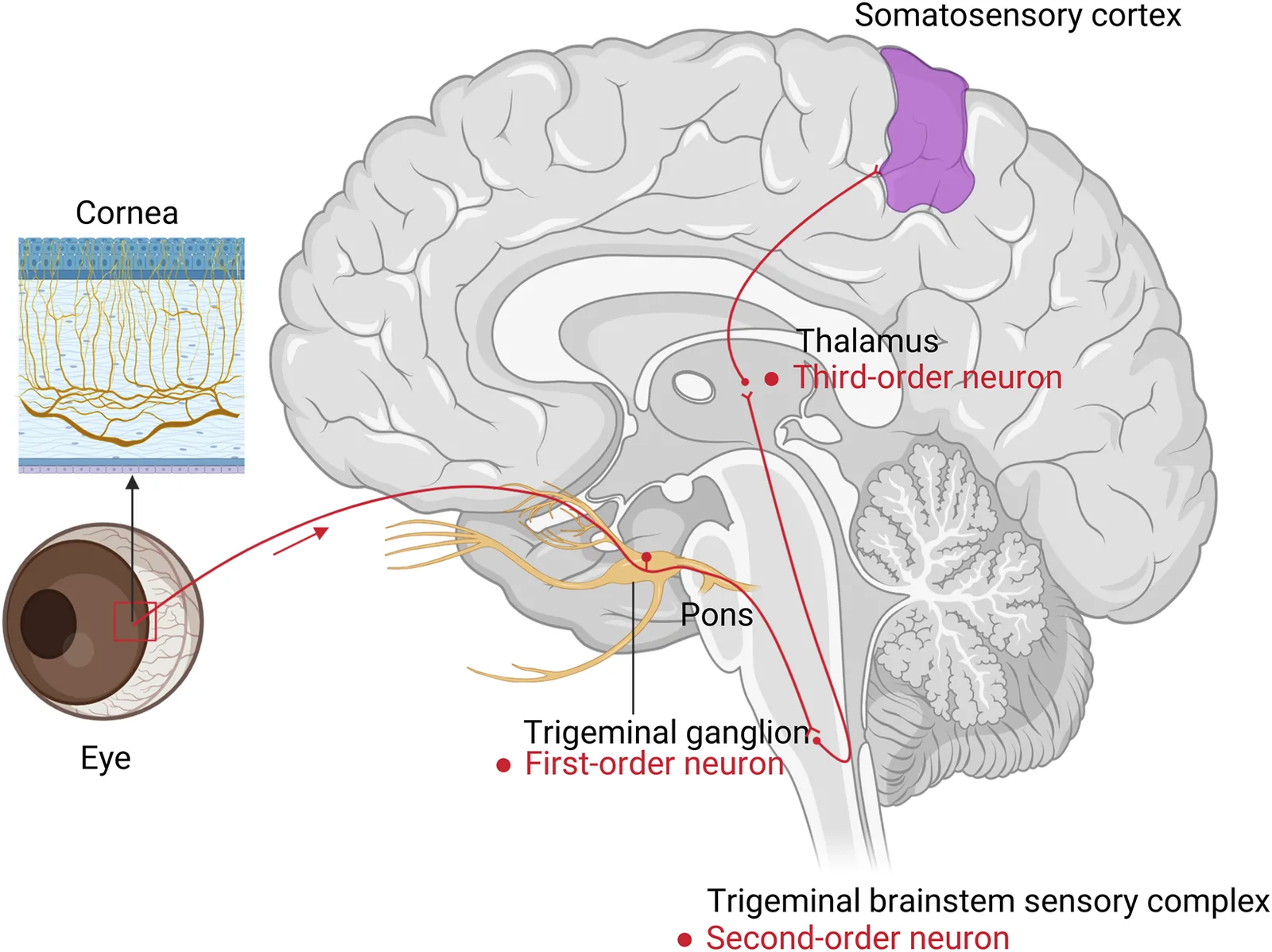
Corneal sensory transmission pathway from peripheral afferents to cortical processing centers. Corneal sensory nerve endings located in the ophthalmic branch (V1) of the trigeminal ganglion (TG) detect mechanical, thermal, and chemical stimuli and transmit signals via pseudounipolar neurons to the trigeminal brainstem sensory complex. Second-order neurons relay the signals to the thalamus, where third-order neurons project to the primary somatosensory cortex, enabling sensory perception. This anatomical pathway provides the structural framework for corneal nociception and serves as the basis for cornea–TG axis regulation discussed in subsequent figures. Created with BioRender.com.

As the transparent tissue located at the anterior surface of the eye, the cornea functions not only as a physical and immunological barrier but also as one of the most densely innervated sensory tissues in the body. The maintenance of corneal transparency, epithelial integrity, hydration balance, wound healing, and sensory perception depends on the coordinated activity of diverse ion channel families expressed in corneal epithelial cells, stromal keratocytes, endothelial cells, and sensory nerve endings (Al-Aqaba et al., 2019); (Figure 1). These include voltage-gated sodium (Na^+^) channels that initiate action potentials, potassium (K^+^) channels that help maintain resting membrane potential and regulate cellular excitability, chloride (Cl^−^) channels that regulate fluid transport and osmotic homeostasis, calcium (Ca^2+^) -permeable channels that mediate intracellular signaling and neurotransmitter release, and a broad array of mechanosensitive and polymodal sensory channels responsible for detecting environmental stimuli (Chalfie, 2009; Yang et al., 2022).

Corneal sensory transduction is mediated by multiple classes of mechanosensitive ion channels that convert physical forces into electrical signals. Members of the transient receptor potential (TRP) family, including TRPV1, TRPA1, and TRPM8, are abundantly expressed in corneal sensory afferents and detect thermal, chemical, osmotic, and inflammatory stimuli, thereby contributing to nociception, tear regulation, and ocular surface homeostasis (Parra et al., 2010; Li et al., 2019; Schecterson et al., 2020). In addition, Piezo channels, particularly Piezo2, function as primary mechanotransducers in trigeminal sensory neurons and corneal mechanonociceptors, mediating the detection of touch and mechanical deformation (Bron et al., 2014; Woo et al., 2015; Fernández-Trillo et al., 2020). Acid-sensing ion channels (ASICs) respond to extracellular acidification associated with tissue injury and inflammation, whereas purinergic P2X receptors, especially P2X3, mediate ATP-dependent sensory signaling following epithelial stress or damage (Wemmie et al., 2013; Yang et al., 2022). Recent studies have also identified mechanosensitive two-pore-domain potassium (K_2P_) channels, including TREK-1, TREK-2, and TRAAK, as important contributors to cellular responses to membrane stretch, osmotic stress, and mechanical force (Honoré, 2007; Brohawn et al., 2014). Together, these channels form an integrated sensory detection system that enables the cornea to perceive and discriminate mechanical, thermal, chemical, and inflammatory stimuli from the external environment.

Corneal sensory information is transmitted through the cornea–trigeminal ganglion (TG) axis, where sensory nerve terminals originating from TG neurons densely innervate the corneal epithelium and stroma. Within this neuroepithelial unit, multiple ion channel classes operate cooperatively to regulate membrane excitability, neurotransmitter release, action potential generation, and nociceptive signaling. Growing evidence indicates that dysregulation of these ion channels contributes to the development of ocular surface disorders, including dry eye disease, neuropathic corneal pain, neurotrophic keratopathy, and chronic ocular inflammation (Yang et al., 2022; Park et al., 2025).

Among the diverse ion channel families involved in corneal sensory physiology, potassium (K^+^) channels occupy a unique position as principal regulators of cellular and neuronal excitability. Unlike excitatory channels that primarily promote membrane depolarization, K^+^ channels serve as key determinants of resting membrane potential, action potential repolarization, firing frequency, and sensory thresholds (Hille, 1978; Goldstein et al., 2005). Consequently, K^+^ channels function as molecular stabilizers or “electrophysiological brakes,” limiting excessive neuronal excitation while preserving physiological sensory responsiveness. Beyond their roles in sensory neurons, K^+^ channels also participate in epithelial wound healing, ion transport, fluid homeostasis, and protection against cellular stress, highlighting their broad importance in maintaining corneal health (Honoré, 2007; Brohawn et al., 2014). Corneal sensitivity is therefore governed by a sophisticated interplay between corneal epithelial cells, stromal cells, endothelial cells, and TG sensory neurons. Within this microenvironment, K^+^ channels act as pivotal mediators of ionic homeostasis and signal integration. By regulating membrane potential and facilitating communication between corneal cells and sensory neurons (Mergler and Pleyer, 2007), these channels establish a functional “perception–conduction–regulation” network along the cornea–TG axis.

Under physiological conditions, corneal K^+^ channels are essential for the sensory conversion of mechanical, chemical, and thermal stimuli. They modulate the activation of TG neurons via neurotransmitter release, thereby ensuring the precise transmission of sensory signals to the central nervous system. Conversely, under pathological conditions, aberrations in K^+^ channel expression or function can disrupt this delicate equilibrium, contributing to the pathogenesis of neuropathic pain, dry eye disease, and other ocular surface disorders. This review systematically examines the classification and distribution of corneal K^+^ channels, their bidirectional regulatory mechanisms with the TG, and their specific roles in disease states, offering a novel perspective on therapeutic strategies for corneal sensory dysfunction.

## 2 Distribution and function of K^+^ channels in the cornea and TG

### 2.1 K^+^ channels in corneal cells

**FIGURE 2:**
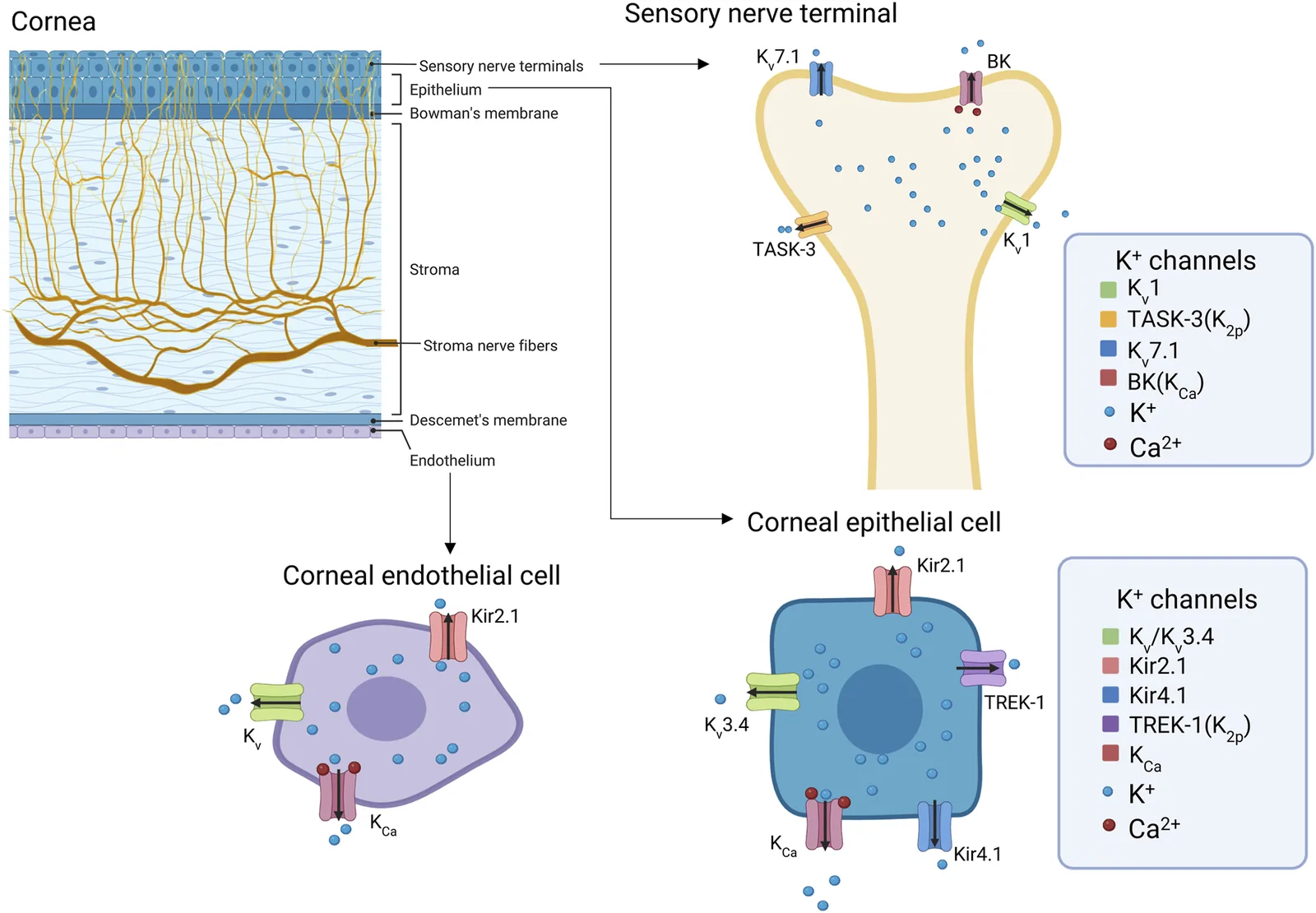
Layer-specific distribution of potassium ion (K^+^) channels in the cornea. This figure illustrates the anatomical organization of the cornea, including the epithelial layer, endothelium, and associated sensory nerve terminals. Distinct K^+^ channel families are differentially distributed across these compartments. In sensory nerve terminals, K_v_ (K_v_1, K_v_7.1), K_2P_ (TASK-3), and K_Ca_ (BK) channels are enriched at the membrane of corneal afferents. In corneal epithelial cells, Kir (Kir2.1, Kir4.1), K_v_3.4, K_Ca_, and K_2P_ (TREK-1) channels are present. In endothelial cells, Kir2.1, K_v_ and K_Ca_ channels contribute to ionic distribution across the cell membrane. This compartmentalized organization highlights the spatial heterogeneity of K^+^ channel expression within the cornea. K_v_, voltage-gated potassium channels; K_2P_, two-pore-domain potassium channels; K_Ca_, Ca^2+^-activated potassium channels; Kir, inward-rectifier potassium channels. Created with BioRender.com.

The cornea is a complex, multilayered structure essential for refractive imaging, intraocular pressure stability, and tectonic protection (Figure 2). Its physiological integrity—comprising the epithelium, stroma, and endothelium—is heavily dependent on K^+^ channels. These channels maintain the resting membrane potential, regulate osmotic pressure, and facilitate the cellular signaling necessary for wound healing and transparency.

Current evidence indicates that K^+^ channels are predominantly expressed in corneal epithelial and endothelial cells, whereas their distribution within stromal keratocytes remains comparatively underexplored (Bonanno, 2012; Anumanthan et al., 2018). Moreover, studies performed in human, mouse, rabbit, and bovine corneas have revealed considerable interspecies differences in channel expression profiles and physiological importance (Yang et al., 2003; Chang et al., 2006; Mergler and Pleyer, 2007; Wang et al., 2014), highlighting potential limitations in translating animal findings to human ocular surface physiology.

#### 2.1.1 Corneal epithelium

The corneal epithelium exhibits the greatest diversity of K^+^ channel expression identified to date. Inward-rectifier potassium (Kir) channels, particularly Kir4.1 (Kcnj10), are highly expressed in murine corneal epithelial cells and contribute to resting membrane potential maintenance, epithelial ion transport, and activation of epidermal growth factor receptor (EGFR)-dependent wound-healing pathways (Lin et al., 2013; Wang et al., 2014; Zhang et al., 2016). Kir2.1 has also been detected in epithelial cells and appears to contribute to membrane stabilization and ionic homeostasis (Rae and Shepard, 2000).

Voltage-gated potassium (K_v_) channels are another major epithelial K^+^ channel family. In the corneal epithelium, K_v_ channels govern developmental processes, including differentiation and programmed cell death (Chang et al., 2006). Specifically, K_v_3.4 has been implicated in ultraviolet-induced apoptosis and regulation of epithelial ion transport, highlighting its role in maintaining epithelial integrity under environmental stress (Wang et al., 2004; Lu, 2006). In addition, Ca^2+^-activated potassium (K_Ca_) channels translate changes in intracellular calcium ([Ca^2+^]i) into electrical signals, primarily to regulate cell volume and osmotic pressure (Faber and Sah, 2003; Thompson-Vest et al., 2006). These are categorized by single-channel conductance into large (BK, K_Ca_1.1), medium (IK, K_Ca_3.1), and small (SK, K_Ca_2.1–2.3) conductance subtypes (Ishii et al., 1997; Orfali and Albanyan, 2023). IK channels are widely expressed within epithelial cells and have been linked to cell proliferation, migration, and tissue repair processes (Ishii et al., 1997; Yang et al., 2013).

More recently, mechanosensitive two-pore-domain potassium (K_2P_) channels have attracted increasing attention. K_2P_ channels (encoded by 15 KCNK genes) provide “leak” currents that determine the background resting membrane potential (Duprat et al., 2007). These are classified into subfamilies based on physiological triggers, such as TWIK (inward rectifiers), TASK (acid-sensitive), and TREK/TRAAK (mechano-sensitive) (Kim and Kang, 2015; Cho et al., 2017; Hughes et al., 2017; Jin et al., 2020). TREK-1 expression has been demonstrated in murine corneal epithelium (Redmon et al., 2025), however, the precise physiological functions of TREK-1 in corneal tissues remain incompletely understood and require further investigation. Given the dense nociceptive innervation of the cornea, it is highly probable that K_2P_ subtypes like TRESK or TREK contribute to the temperature and mechanical sensitivity of corneal afferents (Comes et al., 2021).

#### 2.1.2 Corneal stroma

Compared with epithelial and endothelial layers, knowledge regarding stromal K^+^ channels remain limited. Among stromal potassium channels, IK channels are currently the best-characterized subtype. IK channels is expressed in human corneal stromal cells and regulates fibroblast proliferation, migration, myofibroblast differentiation, and extracellular matrix remodeling, thereby contributing to corneal wound healing and fibrosis (Anumanthan et al., 2018). Pharmacological inhibition of IK channels significantly attenuates corneal scarring and reduces the expression of profibrotic markers such as collagen I and α-smooth muscle actin (α-SMA) (Anumanthan et al., 2018), further characterization of stromal K^+^ channels may provide novel therapeutic targets for corneal scarring and stromal remodeling disorders.

Importantly, systematic cross-species investigations of stromal K^+^ channels are currently lacking, representing a major knowledge gap in the field.

#### 2.1.3 Corneal endothelium

The corneal endothelium maintains corneal transparency through active fluid transport and ionic regulation. Consequently, endothelial cells exhibit abundant expression of K^+^ channels involved in pump function and membrane stabilization, contributing to the active ion transport and resting membrane potential necessary for stromal deturgescence and corneal transparency (Rae and Watsky, 1996; Bonanno, 2012; Amador-Muñoz et al., 2020).

In human corneal endothelial cells, transcriptomic and molecular analyses have confirmed the expression of several cation-activated potassium channels, including SK2 (KCNN2), SK3 (KCNN3), K_Ca_3.1 (KCNN4), and Na^+^-activated K^+^ channels (KCNT2), indicating that K_Ca_-family channels constitute a major component of the endothelial K^+^ channel repertoire (Anumanthan et al., 2018; Amador-Muñoz et al., 2020). These channels likely cooperate to regulate membrane potential, intracellular ion homeostasis, and endothelial pump function.

Electrophysiological studies have further revealed voltage-dependent K^+^ currents in human and animal corneal endothelial cells, supporting the presence of a complex network of K^+^ channels that collectively maintain ionic gradients necessary for transendothelial fluid transport (Rae et al., 1989; Rae and Watsky, 1996).

#### 2.1.4 Species-specific characteristics of corneal K^+^ channels

Although many K^+^ channel families are conserved across species, significant differences exist in their relative abundance and physiological significance.

Comparative studies indicate that the composition and prominence of corneal K^+^ channels differ among species. In human corneal endothelium, SK2 (KCNN2), SK3 (KCNN3), K_Ca_3.1 (KCNN4), and Na^+^-activated K^+^ channels (KCNT2) constitute the major functional repertoire, contributing to membrane potential regulation and endothelial pump function (Anumanthan et al., 2018; Amador-Muñoz et al., 2020). In mouse corneas, Kir4.1 (Kcnj10) predominates in the epithelium and may play a central role in epithelial resting potential and signaling (Rae and Watsky, 1996; Wang et al., 2014). Electrophysiological recordings in rabbit corneal endothelium reveal prominent large-conductance K^+^ currents consistent with BK-like channels (Rae et al., 1989; Rae and Watsky, 1996), whereas bovine corneal endothelium primarily expresses Kir2.1 (Yang et al., 2003). These observations underscore species-specific differences in K^+^ channel composition, emphasizing the need for caution when extrapolating animal data to human physiology.

Despite these observations, direct comparative studies using standardized methodologies remain scarce. Most available data originate from independent studies employing different animal models, experimental conditions, and analytical techniques. Consequently, the field currently lacks a systematic understanding of species-specific K^+^ channel distribution and function, limiting translational interpretation and clinical extrapolation.

#### 2.1.5 Functional classification of corneal K^+^ channels

Collectively, corneal K^+^ channels can be grouped into four major functional categories: (1) Kir channels that stabilize resting membrane potential and regulate epithelial homeostasis; (2) K_v_ channels that control membrane excitability and apoptosis; (3) K_Ca_ channels that couple intracellular Ca^2+^ signaling to proliferation and fluid regulation; and (4) K_2P_ channels that generate background leak currents and contribute to mechanosensory adaptation.

### 2.2 K^+^ channels in trigeminal ganglion

**FIGURE 3:**
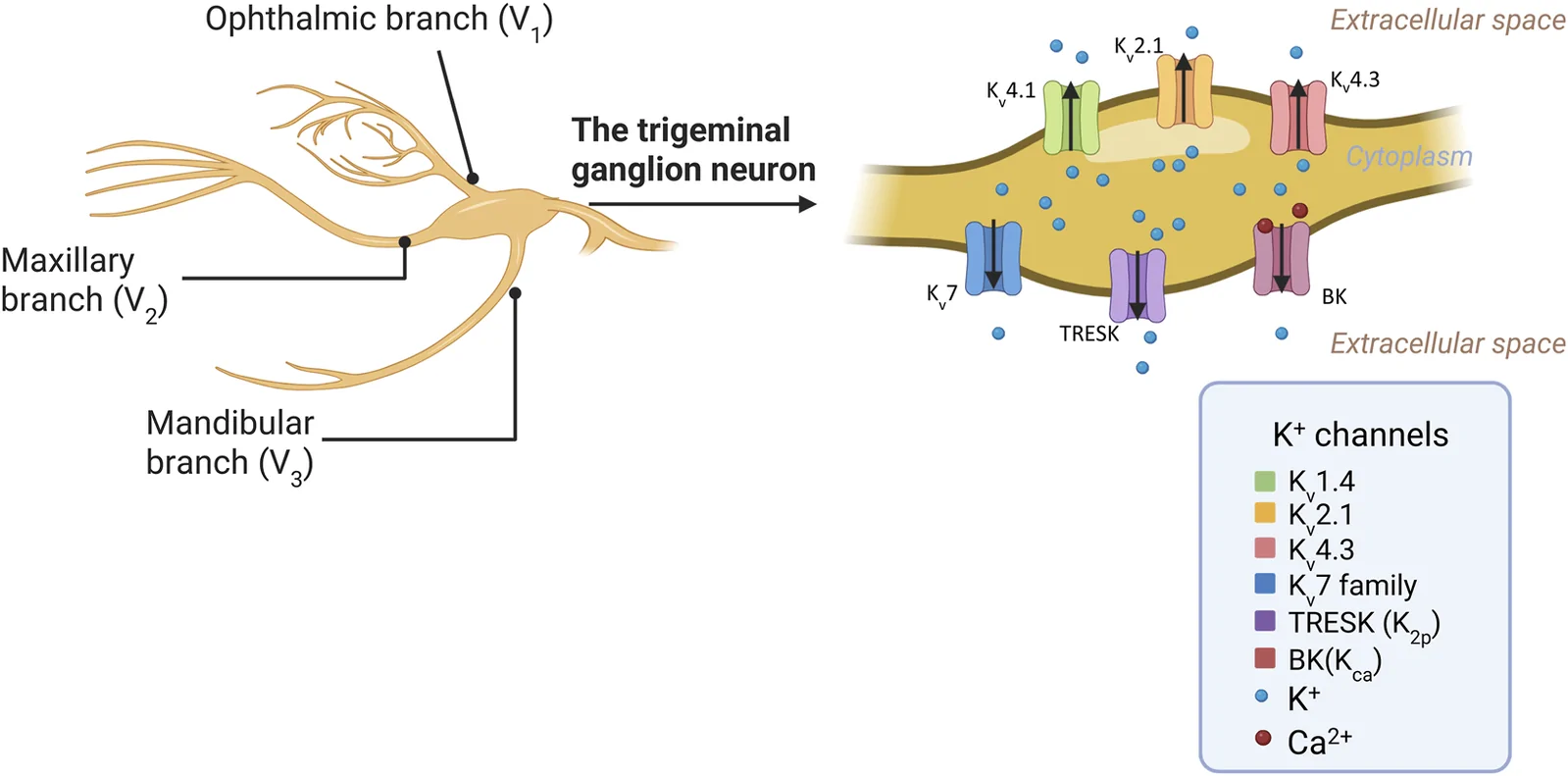
Potassium ion (K^+^) channels in trigeminal ganglion (TG) neurons. This figure illustrates the anatomical organization of TG neurons receiving sensory inputs from the ophthalmic (V1), maxillary (V2), and mandibular (V3) branches of the trigeminal nerve. At the neuronal membrane level, multiple K^+^ channel families are differentially distributed, including K_v_ (K_v_1.4, K_v_2.1, K_v_4.3, and K_v_7), K_2P_ (TRESK), and K_Ca_ (BK). These channels are localized to distinct membrane domains and contribute to the regulation of membrane excitability by shaping ionic fluxes of K^+^ and Ca^2+^ in the intracellular and extracellular environment. The spatial organization of these channel subtypes highlights their coordinated role in controlling action potential dynamics and sensory signal processing within TG neurons. K_v_, voltage-gated potassium channels; K_2P_, two-pore-domain potassium channels; K_Ca_, Ca^2+^-activated potassium channels. Created with BioRender.com.

The trigeminal ganglion (TG) contains the cell bodies of primary sensory neurons that innervate the cornea and serves as the first relay station for corneal sensory information (Belmonte et al., 2004; Yang et al., 2018). TG neurons receive peripheral inputs from corneal mechanoreceptors, polymodal nociceptors, and cold-sensitive afferents and transmit these signals to higher-order sensory centers (Rosenthal and Borsook, 2016; Belmonte et al., 2017) (Figure 3). The excitability of TG neurons is tightly regulated by multiple K^+^ channel families, which control resting membrane potential, action potential generation, firing frequency, and neurotransmitter release (Faber and Sah, 2003; Bean, 2007).

#### 2.2.1 Voltage-gated K^+^ (K_v_) channels

Among these channels, K_v_ channels are major determinants of neuronal excitability (Chow and Leung, 2020). K_v_1.4, K_v_2.1, K_v_4.3, and K_v_7 channels have all been identified in TG neurons and contribute to action potential repolarization and repetitive firing regulation (Abd-Elsayed et al., 2015; Kanda et al., 2021; Zhang et al., 2023; Qi et al., 2025). In particular, K_v_4.3-mediated “A-type” currents play an important role in limiting neuronal hyperexcitability, and reduced K_v_4.3 expression has been associated with peripheral nerve injury and chronic pain conditions (Takeda et al., 2014; Viatchenko-Karpinski et al., 2018). Similarly, activation of K_v_7 channels suppresses neuronal firing and has emerged as a potential therapeutic strategy for neuropathic pain (Wickenden and McNaughton-Smith, 2009; Felix et al., 2025).

#### 2.2.2 Background K^+^ (K_2P_) channels

K_2P_ channels constitute another important class of K^+^ channels in TG neurons. Specifically TRESK, are highly expressed in small-diameter TG neurons and serve as the primary determinants of the resting membrane potential (Yamamoto et al., 2009; Mathie and Veale, 2015). By providing a persistent “leak” current, TRESK channels maintain neuronal stability and raise the threshold required to trigger an action potential. Overexpression of TRESK has been shown to significantly reduce the response of TG neurons to chemical and thermal stimuli (Guo and Cao, 2014). Consequently, activating TRESK has emerged as a promising therapeutic strategy to treat chronic pain and trigeminal neuralgia (Torun et al., 2024). Other K_2P_ channels, including TREK-1, TREK-2, and TASK-3, are expressed in subsets of trigeminal ganglion neurons and contribute to the regulation of background K^+^ conductance, neuronal excitability, mechanosensory processing, and sensory threshold determination (Medhurst et al., 2001; Yamamoto et al., 2009; Royal et al., 2019).

#### 2.2.3 Ca^2+^-activated K^+^ channels (K_Ca_)

K_Ca_ channels further regulate TG neuronal activity by coupling intracellular Ca^2+^ signals to membrane repolarization. Large-conductance BK channels accelerate action potential repolarization and regulate neurotransmitter release, thereby preventing excessive neuronal firing and limiting nociceptive transmission (Kim et al., 2002; Wulf-Johansson et al., 2010). Small- and intermediate-conductance K_Ca_ channels have also been detected in sensory neurons and may contribute to the fine-tuning of neuronal excitability (Bahia et al., 2005; Adelman et al., 2012).

Accumulating evidence suggests that dysregulation of TG K^+^ channels contribute to the pathogenesis of chronic pain disorders. Altered expression and function of K_v_, K_2P_, and K_Ca_ channels have been reported in trigeminal neuropathic pain models. Downregulation of K_v_-mediated outward currents and dysfunction of K_2P_ channels such as TRESK and TREK increase neuronal excitability, whereas impaired K_Ca_ channel activity reduces afterhyperpolarization, collectively contributing to sensory sensitization and persistent pain states (Bahia et al., 2005; Lafrenière et al., 2010; Takeda et al., 2011). Because corneal sensory afferents originate from TG neurons, these channels may also play important roles in ocular surface disorders characterized by sensory dysfunction, including dry eye disease, corneal neuropathic pain, and neurotrophic keratopathy.

Despite significant advances, the distribution and functional roles of K^+^ channels within corneal sensory nerve terminals remain incompletely understood. Future studies combining single-cell transcriptomics, spatial mapping, and electrophysiological approaches will be essential to elucidate how K^+^ channels coordinate signal transmission along the cornea–TG axis and whether these channels represent viable therapeutic targets for ocular surface sensory disorders. A comprehensive overview of K^+^ channel distribution across corneal epithelial, stromal, endothelial, and TG compartments is provided in Table 1.

**TABLE 1:** Distribution and functional roles of K^+^ channel families within the cornea–TG axis.

Channel type	Distribution	Main function	Related role
Corneal cells			
Kir4.1	Epithelial cells	Maintain resting membrane potential, regulate ion homeostasis	Promote epithelial regeneration via EGF/EGFR signaling (Lin et al., 2013; Wang et al., 2014; Zhang et al., 2016)
Kir2.1	Epithelium and endothelium	Contribute to resting membrane potential	Not the major contributor (Rae and Shepard, 2000)
K_Ca_ (BK/IK/SK)	Epithelial cells and endothelium	Regulate osmotic pressure and cell cycle	IK involved in proliferation; may promote angiogenesis (Ishii et al., 1997; Faber and Sah, 2003; Thompson-Vest et al., 2006; Yang et al., 2013; Anumanthan et al., 2018; Amador-Muñoz et al., 2020; Orfali and Albanyan, 2023)
K_v_	Epithelial cells and endothelium	Control cell cycle, apoptosis, ion flux	Kv3.4 mediate UV-induced apoptosis; maintain ion homeostasis (Rae and Watsky, 1996; Wang et al., 2004; Chang et al., 2006; Lu, 2006)
K_2P_ (TREK-1)	Epithelial cells	Regulate resting membrane potential and background K^+^ current	Potential role in sensory neuron regulation (Duprat et al., 2007; Kim and Kang, 2015; Cho et al., 2017; Hughes et al., 2017; Jin et al., 2020; Schecterson et al., 2020; Comes et al., 2021; Redmon et al., 2025)
Corneal sensory neurons			
K_v_1 family	Sensory nerve endings	Counteract depolarization induced by TRPM8	Prevent neuronal overexcitation (Rodolfo et al., 2009)
K_Ca_ (BK)	Sensory nerve endings	Transient hyperpolarization	Inhibit signal transmission under sustained stimulation (Stefan et al., 2007; Robert and Jeremy w, 2009)
K_2P_ (TASK-3)	Sensory nerve endings	Modulate excitability of cold-sensing neurons	Cooperates with TRPM8 to influence pain perception (Morenilla-Palao et al., 2014)
K_v_7.1	Sensory nerve endings	Suppress overactivation	Regulate TRP channel excitability (Ak et al., 2024)
Trigeminal Ganglion (TG)			
K_v_1.4/K_v_2.1/K_v_4.3/K_v_7 family	TG neurons	Shape action potential rise and fall	Regulate neuronal excitability and pain signaling (Takeda et al., 2014; Abd-Elsayed et al., 2015; Viatchenko-Karpinski et al., 2018; Kanda et al., 2021; Zhang et al., 2023; Qi et al., 2025)
K_2P_ (TRESK)	Small-diameter TG neurons	Set resting membrane potential	Reduce neuronal excitability (Yamamoto et al., 2009; Guo and Cao, 2014; Mathie and Veale, 2015; Torun et al., 2024)
K_Ca_ (BK)	TG neurons	Regulate action potential	Pathological downregulation can lead to pain hypersensitivity (Kim et al., 2002; Wulf-Johansson et al., 2010)

## 3 The cornea-TG axis: functional interactions mediated by K^+^ channels

The cornea and trigeminal ganglion (TG) constitute an integrated sensory unit responsible for detecting, transmitting, and processing ocular surface stimuli (Al-Aqaba et al., 2019). While corneal sensory nerve terminals initiate sensory signaling in response to mechanical, thermal, and chemical stimuli, TG neurons integrate and relay this information to central sensory pathways (Meng and Kurose, 2014; González-González et al., 2017; Guerrero-Moreno et al., 2020). K^+^ channels are critical regulators throughout this axis, modulating neuronal excitability, sensory thresholds, signal transmission, and adaptive responses under both physiological and pathological conditions.

### 3.1 K^+^ channels in corneal sensory signal encoding

Corneal nerve endings, primarily Aδ and C fibers, express a diverse array of ion channels that initiate signal transduction (Parra et al., 2010; Meng and Kurose, 2014). While much research has focused on the excitatory influx of Na^+^ and Ca^2+^ via TRP channels (e.g., TRPV1, TRPM8), K^+^ channels provide a critical counter-current that shapes the encoding of sensory signals by regulating receptor potential amplitude, threshold sensitivity, and adaptation (Robert and Jeremy w, 2009; Okada et al., 2015; Ambrosino et al., 2019).

- Action Potential Modulation: K^+^ efflux facilitates membrane repolarization, controlling the frequency and duration of spikes.
- Sub-threshold Encoding: For subtle stimuli (e.g., minor temperature fluctuations), K^+^ channels govern “graded potentials” or membrane resonance. This allows the cornea to encode chronic, low-intensity information without necessarily triggering a full action potential (Puil et al., 1988).
- Pathological Sensitization: In conditions like dry eye disease (DED) or corneal injury, the density and function of these terminals shift. This “end-effector sensitization” is often driven by an imbalance where K^+^ currents are insufficient to counteract the persistent depolarization caused by inflammatory mediators (Pan et al., 2011; Reinach et al., 2015; Guerrero-Moreno et al., 2020).

### 3.2 K^+^ channels in TG sensory signal integration

Once peripheral signals from the cornea reach the TG, their integration and transmission depend on the repertoire of K^+^ channels expressed by TG neurons. Each TG neuron receives convergent inputs from multiple corneal afferent fibers, including mechanonociceptors, polymodal nociceptors, and cold-sensitive fibers. The integration of these inputs determines whether action potentials are propagated centrally, shaping both the magnitude and timing of the signal.

K^+^ channels play a central role in this signal integration.

- K_v_ channels regulate action potential repolarization and interspike intervals, allowing TG neurons to filter out low-level or repetitive stimuli and prevent excessive firing. Alterations in K_v_ channel expression, as observed in neuropathic pain models, lead to enhanced excitability and aberrant signal amplification (Feng et al., 2013; Viatchenko-Karpinski et al., 2018).
- K_2P_ channels, such as TRESK, TREK, and TASK subtypes, generate background leak currents that stabilize the resting membrane potential. By setting firing thresholds, K_2P_ channels determine which convergent inputs can trigger action potentials, effectively acting as a gate for sensory integration (Dobler et al., 2007; Enyedi and Czirják, 2015). Species-specific differences in K_2P_ channel expression may influence signal processing, with certain subtypes more prominently expressed in human TG neurons compared with rodent models (Andres-Enguix et al., 2012; Lengyel et al., 2020).
- K_Ca_ channels including BK, SK, and IK, mediate afterhyperpolarization following action potentials (Faber and Sah, 2003). This mechanism provides negative feedback that modulates spike frequency and temporal summation of convergent inputs, fine-tuning sensory signal integration before transmission to central pathways.

Together, these K^+^ channel families allow TG neurons to integrate a variety of sensory inputs, control firing probability, and encode information about stimulus intensity, duration, and modality. Dysfunction in any of these channels disrupts integration, potentially leading to hyperexcitability and abnormal pain perception in conditions such as trigeminal neuropathic pain.

### 3.3 Cornea-to-TG communication

Peripheral injury or inflammation triggers the release of neurotransmitters and inflammatory mediators from corneal epithelial cells, which act on TG neurons to modulate K^+^ channel activity, thereby linking tissue damage to altered neuronal excitability.

The major inflammatory mediators involved in this cornea–TG signaling axis and their effects on K^+^ channels in TG neurons are summarized in Table 2.

**TABLE 2:** Representative mediators regulating K^+^ channel activity in TG neurons.

Mediator	Receptor	Effect on K^+^ channels	Functional outcome
ATP	P2X3/P2Y	Activates BK channels via [Ca^2+^]_i_	Promotes repolarization; modulates firing frequency (Masterson and Durham, 2010; Oswald et al., 2012)
Glutamate	NMDA	Inhibits K_v_2.1 channels	Prolongs action potential duration; increases signal persistence (Mulholland et al., 2008)
PGE_2_	EP Receptors	Enhances NMDA-mediated excitability	Lowers the activation threshold for nociceptors (Patwardhan et al., 2008)

### 3.4 TG-to-cornea feedback and Pathological Sensitization

The cornea–TG axis operates as a bidirectional sensory loop. Peripheral inflammation in the cornea can trigger “retrograde signaling” that downregulates K^+^ channels (like K_v_4.3 and TRESK) in the TG, leading to central sensitization (Takeda et al., 2014; Viatchenko-Karpinski et al., 2018). Conversely, a hyperexcitable TG can lead to “neurogenic inflammation” at the corneal surface via the release of neuropeptides from efferent branches (Mantelli et al., 2010; Lasagni Vitar et al., 2022). This bidirectional communication creates a self-reinforcing loop that can maintain corneal hypersensitivity and chronic ocular pain even after the initial injury has resolved. Such maladaptive feedback underscores the central role of K^+^ channel regulation in controlling neuronal excitability and sensory homeostasis along the cornea–TG pathway.

## 4 K^+^ channels in ocular surface disorders

The maintenance of ocular surface homeostasis requires coordinated regulation of epithelial integrity, stromal remodeling, immune responses, and sensory nerve function (Chen et al., 2022). Increasing evidence suggests that K^+^ channels are not merely passive regulators of membrane potential but active participants in multiple signaling pathways involved in tissue repair, inflammation, fibrosis, and sensory processing (Giblin et al., 2016). Dysregulation of K^+^ channel activity can alter ionic homeostasis, intracellular Ca^2+^ signaling, and downstream molecular pathways, ultimately contributing to corneal epithelial defects, dry eye disease (DED), corneal neuropathic pain (CNP), and other ocular surface disorders (Galor et al., 2015; Reinach et al., 2015). Importantly, the pathological consequences of K^+^ channel dysfunction are frequently mediated through interconnected signaling pathways rather than isolated channel-specific effects. Current evidence indicates that K^+^ channels participate in EGFR-dependent epithelial repair, TGF-β-mediated fibrotic remodeling, inflammasome-associated inflammatory signaling, and TRP channel-dependent sensory transduction, thereby linking ionic homeostasis to structural and functional alterations of the ocular surface.

### 4.1 K^+^ channels in corneal epithelial injury and repair

Corneal wound healing is a highly coordinated process involving epithelial migration, proliferation, differentiation, extracellular matrix remodeling, and restoration of sensory nerve function (Ljubimov and Saghizadeh, 2015). These events can be broadly divided into three overlapping phases: (1) initiation and migration, (2) proliferation and tissue reconstruction, and (3) remodeling and maturation (Wilson et al., 2001). K^+^ channels participate in each phase by regulating membrane potential, Ca^2+^ influx, and intracellular signaling pathways that govern cellular behavior.

#### 4.1.1 Regulation of epithelial migration and proliferation

Among the K^+^ channels involved in epithelial repair, Kir4.1 has emerged as an important regulator of epithelial homeostasis. Under physiological conditions, Kir4.1 contributes to maintenance of the resting membrane potential and local ionic microenvironment required for epithelial cell survival and proliferation (Zhang et al., 2016; Chen et al., 2022). Following corneal injury, modulation of Kir4.1 activity influences membrane polarization states and alters epidermal growth factor receptor (EGFR)-dependent signaling pathways.

Experimental studies have demonstrated that reduced Kir4.1 activity is associated with membrane depolarization and enhanced EGFR phosphorylation, leading to activation of downstream ERK and PI3K/Akt signaling cascades (Zhang et al., 2016). These signaling pathways promote epithelial cell migration and proliferation, thereby facilitating wound closure. Furthermore, upstream regulators such as Caveolin-1 (Cav1) and miR-205 have been shown to influence Kir4.1 expression and function, suggesting the existence of a Cav1–Kir4.1–EGFR signaling axis involved in epithelial repair (Lin et al., 2013; Zhang et al., 2016).

Although most mechanistic evidence has been obtained from murine models and cultured epithelial cells, these findings collectively support the concept that K^+^ channels participate in wound healing by linking changes in membrane potential to growth factor-mediated cellular responses.

#### 4.1.2 Regulation of inflammation, fibrosis, and tissue remodeling

In addition to epithelial repair, K^+^ channels contribute to stromal remodeling following injury. K_Ca_3.1 channels are expressed in multiple corneal cell populations, including epithelial cells, stromal fibroblasts, keratocytes, and endothelial cells, suggesting that their biological effects extend beyond a single cellular compartment (Ledoux et al., 2008; Anumanthan et al., 2018).

Activation of K_Ca_3.1 promotes membrane hyperpolarization and increases the electrochemical driving force for Ca^2+^ entry. Sustained intracellular Ca^2+^ signaling subsequently enhances TGF-β-dependent pathways that regulate fibroblast activation, myofibroblast differentiation, and extracellular matrix production (Chou et al., 2008; Roach et al., 2015; Anumanthan et al., 2018). Excessive activation of these pathways may contribute to corneal fibrosis, stromal opacity, and scar formation.

Experimental studies have demonstrated that pharmacological inhibition of overactive K_Ca_3.1 channel with TRAM-34 attenuates corneal edema, neovascularization, and fibrotic remodeling in injury models (Yang et al., 2013). These findings suggest that K_Ca_3.1 represents a potential therapeutic target for limiting pathological tissue remodeling while preserving physiological wound healing.

Collectively, current evidence indicates that different K^+^ channel subtypes regulate distinct stages of corneal repair. Whereas Kir4.1 primarily influences epithelial migration and proliferation through growth factor-dependent signaling, K_Ca_3.1 contributes more prominently to inflammatory and fibrotic responses through modulation of intracellular Ca^2+^ signaling.

### 4.2 K^+^ channel dysfunction in dry eye disease and corneal neuropathic pain

Dry eye disease (DED) is increasingly recognized as a disorder involving both ocular surface inflammation and neurosensory abnormalities. Persistent inflammatory stimulation can alter the electrophysiological properties of corneal sensory neurons and trigeminal ganglion (TG) neurons, resulting in peripheral and central sensitization (Galor et al., 2015; Dieckmann et al., 2022). Dysfunction of K^+^ channels is increasingly recognized as a key contributor to these pathological changes.

#### 4.2.1 Peripheral sensitization at corneal nerve endings

Corneal sensory afferents normally maintain a balance between excitatory currents mediated by channels such as TRPV1, TRPA1, and TRPM8 and inhibitory currents mediated by various K^+^ channels (Belmonte et al., 2004; Luu et al., 2024). This balance ensures appropriate sensory thresholds and prevents excessive neuronal firing.

During DED, as mentioned before, inflammatory mediators including cytokines, prostaglandins, and ATP enhance excitatory channel activity while simultaneously reducing K^+^ channel-mediated inhibitory control (Mulholland et al., 2008; Patwardhan et al., 2008; Masterson and Durham, 2010; Oswald et al., 2012). The resulting shift toward membrane depolarization lowers activation thresholds and increases neuronal responsiveness to mechanical, thermal, and osmotic stimuli. Consequently, sensory neurons become hypersensitive, contributing to ocular discomfort, burning sensations, and pain.

Several K^+^ channel families appear to participate in this process. KCNQ (K_v_7) channels generate M-type currents that suppress repetitive firing (Abd-Elsayed et al., 2015), whereas TASK-family K_2P_ channels provide background leak currents that stabilize resting membrane potential (Lengyel et al., 2021; Liao et al., 2024). Emerging evidence also suggests that KCNK5 may participate in inflammatory signaling pathways associated with epithelial stress responses and pyroptosis (Liao et al., 2024). Although further studies are needed, these observations indicate that K^+^ channel dysfunction may contribute not only to altered excitability but also to amplification of inflammatory signaling at the ocular surface.

#### 4.2.2 Central sensitization in TG neurons

Persistent nociceptive input originating from the ocular surface can induce maladaptive plasticity within the TG and central sensory pathways, contributing to central sensitization and chronic pain states (Galor et al., 2015). This transition from peripheral sensitization to central sensitization is considered a critical step in the development of chronic ocular pain and corneal neuropathic pain.

Among the K^+^ channels expressed in TG neurons, TRESK has received particular attention because of its role in generating background leak currents that stabilize resting membrane potential. Reduced TRESK activity diminishes this stabilizing influence and promotes neuronal hyperexcitability (Guo and Cao, 2014; Lengyel et al., 2021). Similar effects have been observed following downregulation of K_v_ and KCNQ channels, which normally contribute to repolarization and suppression of repetitive firing (Passmore et al., 2003; Jones et al., 2026).

Cold-sensitive corneal afferents provide an illustrative example of these mechanisms. These neurons co-express TRPM8 together with several K^+^ channel subtypes, including K_v_1 family channels and TASK-3 (Madrid et al., 2009; Fakih et al., 2020). Under physiological conditions, K^+^ currents counterbalance TRPM8-mediated depolarization and restrict excessive cold-evoked firing (Rodolfo et al., 2009). Reduction of K^+^ conductance weakens this inhibitory constraint and may contribute to cold hypersensitivity and allodynia commonly observed in patients with corneal neuropathic pain.

Taken together, current evidence suggests that impaired K^+^ channel function contributes to both peripheral and central sensitization by facilitating membrane depolarization, lowering firing thresholds, and amplifying nociceptive signaling.

### 4.3 Integrated pathophysiological implications of K^+^ channel dysfunction

Collectively, the evidence discussed above suggests that K^+^ channel dysfunction contributes to ocular surface disease through interconnected rather than isolated mechanisms. Altered K^+^ conductance affects membrane potential, intracellular Ca^2+^ homeostasis, and downstream signaling pathways, thereby influencing epithelial repair, inflammatory responses, fibrotic remodeling, and sensory processing.

Importantly, these effects extend across the entire cornea–TG axis. Through their interactions with signaling networks involved in tissue repair, inflammation, and neuronal excitability, K^+^ channels function as key regulators linking structural and sensory components of ocular surface disease. Consequently, disruption of K^+^ channel-mediated homeostasis may contribute simultaneously to impaired wound healing, chronic inflammation, fibrosis, and sensory sensitization, highlighting their central role in maintaining ocular surface function. The therapeutic implications of these mechanisms across the cornea–TG axis are summarized in Table 3.

**TABLE 3:** Mechanism-based modulation of K^+^ channels in ocular surface disease.

Target	Channel type	Mechanistic modulation	Functional effect	Pathophysiological outcome
Kir4.1	Kir	Agonist/Modulator	Restores membrane potential and supports EGFR-dependent signaling	Accelerate epithelial wound healing
KCa3.1 (IK)	KCa	Antagonist	Modulates Ca^2+^-dependent signaling and reduces fibroblast activation	Prevent corneal fibrosis and angiogenesis
KCNQ	Kv	Activator	Stabilizes membrane excitability and suppresses repetitive firing	Reduce firing frequency in neuropathic pain
TRESK	K2P	Activator	Restores background K^+^ conductance and stabilizes resting membrane potential	Restore resting potential in sensitized TG neurons

## 5 Conclusion and future perspectives

The cornea and trigeminal ganglion (TG) constitute a highly specialized sensory and protective unit, where K^+^ channels serve as the fundamental regulators of the “perception-conduction-regulation” axis. Studies to date have delineated distinct roles for Kir4.1 in EGFR-mediated epithelial healing, K_Ca_3.1 in TGF-β-driven fibrotic remodeling, and K_2P_/K_v_ channels in stabilizing neuronal excitability and controlling nociceptive signaling. Collectively, these findings highlight the integral contribution of K^+^ channels to ocular surface homeostasis and sensory function.

### 5.1 Current limitations

Despite this progress, several critical limitations constrain mechanistic and translational interpretations. Most evidence is derived from animal models, including mouse, rabbit, and bovine corneas, with limited direct validation in human tissues. Species-specific differences in channel expression and functional dominance complicate the extrapolation of findings. Experimental designs, disease models, and channel isoform characterization vary widely across studies, impeding robust cross-study comparisons. Furthermore, many reports lack clear cell-type resolution, obscuring the compartment-specific contributions of epithelial, stromal, endothelial, and neuronal K^+^ channels.

Methodological constraints also pose challenges. Standardized, physiologically relevant models that recapitulate the cornea–TG axis are scarce, and highly selective pharmacological tools for individual K^+^ channel subtypes are limited. Additionally, most studies are cross-sectional rather than longitudinal, restricting insights into the dynamic regulation of K^+^ channels during disease progression, injury response, and wound healing. These limitations collectively underscore the need for more rigorous, human-relevant, and mechanistically precise approaches to strengthen causal and translational conclusions.

### 5.2 Translational perspectives

K^+^ channels represent promising therapeutic targets due to their capacity to modulate multiple pathogenic processes simultaneously. Targeting Kir4.1 may accelerate epithelial repair, while K_Ca_3.1 inhibition could mitigate TGF-β-driven fibrosis. Activation of neuronal K^+^ channels, including KCNQ, TRESK, and TASK-family subtypes, has the potential to restore inhibitory control, reduce hyperexcitability, and alleviate chronic ocular pain. However, systemic side effects remain a concern given the ubiquitous expression of these channels, emphasizing the importance of localized ocular delivery strategies, such as topical formulations, subconjunctival inserts, or biomaterial-based platforms.

### 5.3 Future research directions

Future investigations should focus on integrated, multi-level analyses combining molecular, cellular, and systems approaches. High-resolution, cell-type specific mapping of K^+^ channel expression in human corneal layers and TG neurons will be essential to resolve species differences and compartment-specific functions. Standardized, physiologically relevant models that recapitulate the cornea–TG axis, coupled with selective pharmacological or genetic tools, will enable mechanistic dissection of channel function. Longitudinal studies are also warranted to elucidate dynamic changes in channel activity during disease progression, wound healing, and therapeutic interventions.

In parallel, precision medicine approaches may identify patients with genetic variations in K^+^ channel genes who are predisposed to severe dry eye disease or corneal neuropathic pain. Combining these insights with emerging gene therapy or localized pharmacological strategies could enable individualized modulation of channel activity to restore homeostasis across the cornea–TG axis.

In summary, while substantial gaps and methodological challenges remain, K^+^ channels are no longer passive “leak” pathways but are recognized as dynamic regulators of ocular surface physiology and pathology. Addressing the current limitations through human-relevant, mechanistically precise, and longitudinal studies will be critical for translating these insights into effective therapies for ocular surface diseases and chronic sensory dysfunction.
